# Role of *Rhizobium* endoglucanase CelC2 in cellulose biosynthesis and biofilm formation on plant roots and abiotic surfaces

**DOI:** 10.1186/1475-2859-11-125

**Published:** 2012-09-12

**Authors:** L Rivera, Jose I Jiménez-Zurdo, R Rivas, F Dazzo, E Velázquez, E Martínez-Molina, Ann M Hirsch, Pedro F Mateos

**Affiliations:** 1 Departamento de Microbiología y Genética and CIALE, Universidad de Salamanca, Salamanca, Spain; 2 Estación Experimental del Zaidín, CSIC, Granada, Spain; 3 Department of Microbiology and Molecular Genetics, Michigan State University, Lansing, USA; 4 Department of Molecular, Cell and Developmental Biology and Molecular Biology Institute, University of California-Los Angeles, Los Angeles, USA; 5 LOEWE Research Center for Synthetic Microbiology (SYNMIKRO), Marburg, Germany

**Keywords:** Cellulose biosynthesis, *Rhizobium*, Cellulases, Biofilm, Symbiosis

## Abstract

**Background:**

The synthesis of cellulose is among the most important but poorly understood biochemical processes, especially in bacteria, due to its complexity and high degree of regulation. In this study, we analyzed both the production of cellulose by all known members of the *Rhizobiaceae* and the diversity of *Rhizobium celABC* operon predicted to be involved in cellulose biosynthesis. We also investigated the involvement in cellulose production and biofilm formation of *celC* gene encoding an endoglucanase (CelC2) that is required for canonical symbiotic root hair infection by *Rhizobium leguminosarum* bv. trifolii.

**Results:**

ANU843 *celC* mutants lacking (ANU843ΔC2) or overproducing cellulase (ANU843C2^+^) produced greatly increased or reduced amounts of external cellulose micro fibrils, respectively. Calcofluor-stained cellulose micro fibrils were considerably longer when formed by ANU843ΔC2 bacteria rather than by the wild-type strain, in correlation with a significant increase in their flocculation in batch culture. In contrast, neither calcofluor-stained extracellular micro fibrils nor flocculation was detectable in ANU843C2^+^ cells. To clarify the role of cellulose synthesis in *Rhizobium* cell aggregation and attachment, we analyzed the ability of these mutants to produce biofilms on different surfaces. Alteration of wild-type CelC2 levels resulted in a reduced ability of bacteria to form biofilms both in abiotic surfaces and *in planta*.

**Conclusions:**

Our results support a key role of the CelC2 cellulase in cellulose biosynthesis by modulating the length of the cellulose fibrils that mediate firm adhesion among *Rhizobium* bacteria leading to biofilm formation. *Rhizobium* cellulose is an essential component of the biofilm polysaccharidic matrix architecture and either an excess or a defect of this “building material” seem to collapse the biofilm structure. These results position cellulose hydrolytic enzymes as excellent anti-biofilm candidates.

## Background

Symbioses between diazotrophic rhizobia and legume plants are of critical agronomic and environmental importance, making crop production possible in nitrogen-limited soils without fertilizer supply. Rhizobia grow as free-living organisms, but can also induce and colonize root nodules in legume plants thereby establishing a partnership that benefits both organisms. This process begins when flavonoids secreted by the plant induce *Rhizobium nod* genes, which are involved in the synthesis and secretion of lipo-chitooligosaccharide signal molecules, known as Nod factors. In response, plant root hairs deform and exhibit a typical marked curling to facilitate bacteria penetration. The interaction continues with the initiation of the root nodule, where bacterial cells are released into the host cells. Eventually, upon a morphological differentiation into bacteroids, bacteria fix atmospheric dinitrogen into ammonia. Among the many factors involved in development of an effective symbiosis between rhizobia and their host plants, those associated with adherence and colonization of bacteria on the surface of roots and the root hair tip —a key stage for the subsequent entry into the plant— have not been fully characterized. Root attachment and colonization by rhizobia follow the two-phase sequence of events previously described for bacteria in general [1]. Several bacterial proteins, such as adhesins, and flagellar proteins [2,3], have been proposed to be important factors for the early reversible, specific binding events, whereas bacterial cell-surface polysaccharides are the main components involved in the later, irreversible attachment stages. Some of these cell surface components include exopolysaccharide, lipopolysaccharides, and cyclic β-1,2-glucans [4-6], but mainly cellulose fibrils, which mediate firm adhesion of the bacteria to root hairs and anchor bacteria to the root surface [1,7,8]. Hapten-inhibitable root hair lectins are also involved in this dynamic attachment process [9-12].

Attachment of bacteria to a substratum is the initial step in biofilm formation, which is followed by the establishment of micro colonies by clonal propagation, and final maturation into three-dimensional structures that are covered by exopolymer and other matrix materials [3,5,13,14]. These bacterial three-dimensional structures are of considerable biotechnological importance due to their implications not only in bacterial colonization of abiotic surfaces with an economic value, but also in almost all pathogenic infections, making them recalcitrant due to their multidrug resistance. Initially, the proposed function for cellulose in bacteria was not linked to biofilm formation. However, recent studies have revealed that some species of the family *Enterobacteriaceae* (e.g., *Citrobacter* spp., *Enterobacter* spp., and *Klebsiella* spp.) produce cellulose as a crucial component of the bacterial extracellular matrix (reviewed in 25). In wild-type rhizobia, this polysaccharide of industrial interest is produced in flocculating batch cultures, some constitutively, others at early stationary phase [15], and upon contact with the roots of the host plant [16]. Cellulose has been proposed to be required for optimal infection of long root hairs [17] and biofilm cap formation at pH 6.5 and 7.5 [18]. Other studies have shown that cellulose is involved in anchoring the pathogen *Agrobacterium tumefaciens* to plant tissue, thereby affecting virulence [19]. In plant culture, *Rhizobium leguminosarum* bv. trifolii polymerizes β-1-4 glucose residues into cellulose micro fibrils that are anchored to the roots of the host plant *Trifolium repens*[1,8].

Biosynthesis of cellulose, a plant process required for cell wall growth, has also been described in some bacteria and even in one group of animals, the urochordates [20]. Although significant in the medical, agricultural and ecological context, the biological role of cellulose biosynthesis and its regulation has not been widely studied in bacteria, apart from *Gluconacetobacter xylinum* (formerly called *Acetobacter xylinum*) [21]. However, it is widespread in Gram negative and Gram positive bacteria and also in some species of cyanobacteria [15,22-25].

In both bacteria and plants, two proteins have been identified as directly involved in the biosynthesis of cellulose: CesA and Korrigan in plants, and CelA and CelC in bacteria [23,26]. CelA and CesA have homology with glycosyl transferases while CelC and Korrigan are homologous to endoglucanases. Orthologs of the putative cellulose biosynthesis genes *celABC* have been found in a region of the chromosome that is involved in cellulose biosynthesis among a variety of bacteria that synthesize cellulose [16,19,27,28].

The first gene in the operon is *bcsA* (bacterial cellulose synthesis), also named *acsA* or *celA*. It encodes a cellulose synthase that harbors a β-glycosyltransferase 2 domain and binds the substrate UDP-glucose. It is also the longest and best-conserved protein encoded by the *bcs* operon among diverse species [29]. The second gene, *bcsB* (synonyms: *acsB*, *celB*), encodes a cyclic diguanylic acid (c-di-GMP) binding protein and is less conserved. The last gene, *bcsZ* (also called *celC* in *A. tumefaciens* and *R. leguminosarum* bv. trifolii) has been shown to encode a cellulase (family 8 glycosyl hydrolase), which is present in all cellulose-producing species. *Rhizobium celC* is also located in the *celABC* operon [16]. BcsZ homologs are also located in the cellulose biosynthesis operons of enterobacterial species, but outside yet adjacent to this operon in several *Gluconacetobacter xylinum* strains [30]. Other genes such as *bcsC* and *bcsD*, both homologs to *Rhizobium celE* gene, are required for *in vivo* but not for *in vitro* cellulose biosynthesis [31].

The lack of cellulose production in *celA* or *celB* mutants of *R. leguminosarum* bv. trifolii did not affect the ability of these bacteria to nodulate clover under controlled laboratory conditions [16]. The postulated involvement of the *Agrobacterium* CelC cellulase enzyme in bacterial cellulose biosynthesis is to incorporate a lipid –linked oligosaccharide intermediate into cellulose [32], but this biochemical function has not been definitely established. *R. leguminosarum* bv. trifolii ANU843 CelC2 has high substrate specificity for amorphous forms of cellulose (e.g., carboxymethyl cellulose, CMC) and does not degrade other polysaccharides tested so far [28].

Studies using purified enzyme, CelC2 knockout and overproducing derivative strains of *R. leguminosarum* bv. trifolii ANU843 show that this cellulase isozyme is essential for primary infection of its symbiotic white clover host (*Trifolium repens*), being required for the localized tip erosion of the root hair wall allowing the bacteria to breach this host barrier and initiate the infection process [28,33]. CelC2 has been also shown to be involved in bacterial release from infection threads into nodule cells [34]. Evaluation of the currently defined genomes of rhizobia indicated genes coding for putative cellulases represented by a diversity of glycosyl hydrolase families [28]. The *celC* gene is widely conserved among *Rhizobium* species [35], consistent with its involvement in fundamental processes for survival in the environment. In this work, we provide evidence for a key role of the CelC2 cellulase for optimal cellulose micro fibrils biosynthesis and biofilm formation.

## Results

### Cellulose production is widespread among rhizobia

**Figure 1:**
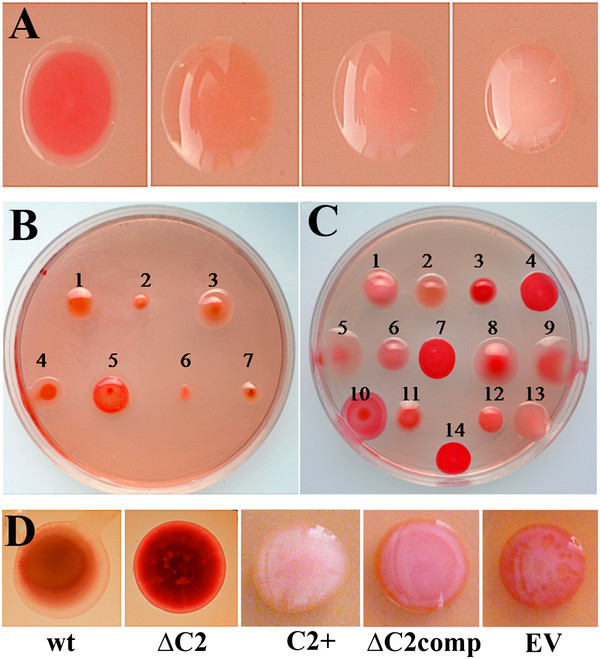
**Bacterial colonies grown on YMA containing Congo red.A**) Different color intensities of Congo red binding in different strain, from left to right: *R. cellulosilyticum* ALA10B2 ^T^ (+++), *R. leguminosarum* bv. trifolii ANU843 (++), *R. hainanense* I66^T^ (+), and *E. kostiense* LMG 19227 ^T^ (w). **B**-**C**) Congo red uptake by colonies of root-nodule legume symbionts: (**B**) representative type strains of different genera: 1. *R. phaseoli* ATCC 14482^T^*,* 2. *E. meliloti* ATCC 9930^T^*,* 3. *M. loti* ATCC 33669^T^, 4. *B. elkanii* LMG 6134^T^, 5. *P. trifolii* PETPO2 ^T^*,* 6. *A. caulinodans* ORS 571^T^*,* 7. *D. neptuniae* J1^T^ and (**C**) representative type strains of genus *Rhizobium*: 1. *R. hainanense* I66^T^, 2. *R. leguminosarum* ATCC10004^T^, 3. *R. galegae* ATCC 43677^T^, 4. *R. etli* CFN 42^T^, 5. *R. lusitanum* P1-7^T^, 6. *R. loessense* CCBAU 7190B^T^, 7. *R. giardinii* H152^T^, 8. *R. mongolense* USDA 1844^T^, 9. *R. indigoferae* CCBAU 71042^T^, 10. *R. tropici* CIAT 899^T^, 11. *R. yanglingense* CCBAU 71623, 12. *R. huautlense* SO2^T^, 13. *R. gallicum* R602sp^T^, 14. *R. cellulosilyticum* ALA10B2^T^ and (**D**) wild-type strain of *Rhizobium leguminosarum* bv. trifolii ANU843 (wt) and its derivatives ANU843ΔC2 (ΔC2), ANU843C2^+^ (C2+), ANU843ΔC2 complemented (ΔC2comp) and ANU843EV (EV).

Currently, the known diversity of bacteria that can establish symbiotic nitrogen fixing root nodules with legumes, mostly resides within the order *Hyphomicrobiales*, which includes about 83 species distributed in different families and genera. We examined most of the official type strains of each of these taxa for cellulose production by Congo red staining, and all were found to be positive to varying degrees (Figure 1B-C, Additional file 1).

A database search revealed that all the currently defined *Rhizobiaceae* genomes possess genes coding for cellulose synthase belonging to the Glycosyl Transferase family 2 (GT2). Interestingly, these GT2 coding genes are located near endoglucanase *celC* homologs (belonging to Glycosyl Hydrolase family 8) forming the *celABC* operon or near cellulase genes from Glycosyl Hydrolase family 26, forming a potential operon that contains a cellulose synthase associated with a cellulase and another hypothetical protein of unknown function, that we have named *celIJK*. This putative operon has homologs in all currently available genomic sequences of *Rhizobiaceae* representatives (Additional file 2). There are several organisms that share both cellulose production operons, and in *Agrobacterium tumefaciens* wild-type strain C58, *celABC* and *celIJK* are closely located in the genome.

We have sequenced the *celABC* genes from *R. leguminosarum* bv. trifolii ANU843 (GeneBank accession no. JN180924, *celA*; JN180925, *celB*; AJ561043, *celC*). Their nucleotide sequence and organization are similar and highly conserved among various *Rhizobiaceae* members. Orthologs of this operon are also found in other legume-nodulating bacteria (Additional file 3).

### CelC2 cellulase is involved in cellulose microfibril formation and elongation

**Figure 2:**
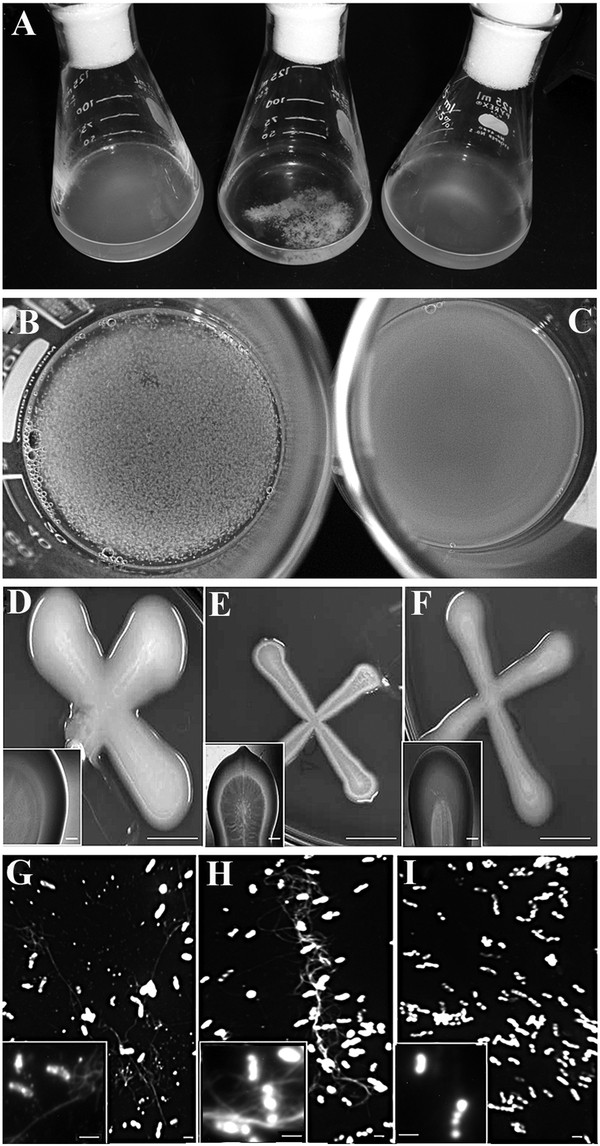
**From left to right, strain ANU843 (wild type) and its derivatives ANU843ΔC2 and ANU843C2**^**+**^**.** (**A**) Batch cultures in stationary phase in YMB shaken at 180 rpm. (**B**-**C**) ANU843ΔC2 flocs after incubation 2h at 37°C in PCA buffer pH 5 (**B**) and containing 10 U/ml *Trichoderma viride* commercial cellulase (**C**). (**D**) Line streak colonies grown on TY (bar = 1cm). The insert in the lower left corner is an enlargement of the images (bar = 1 mm). (**G-I**) Calcofluor staining showed the presence of micro fibrils (bar = 1.0 μm) in wild type ANU843 (**G**), ANU843ΔC2 (**H**) and ANU843C2^+^ (**I**). Inserts at the lower left corners show representative cells at higher magnification. In the insert images note the accumulation of bright fluorescent target at both cell poles separated by reduced fluorescence intensity midway between the cell poles.

**Figure 3:**
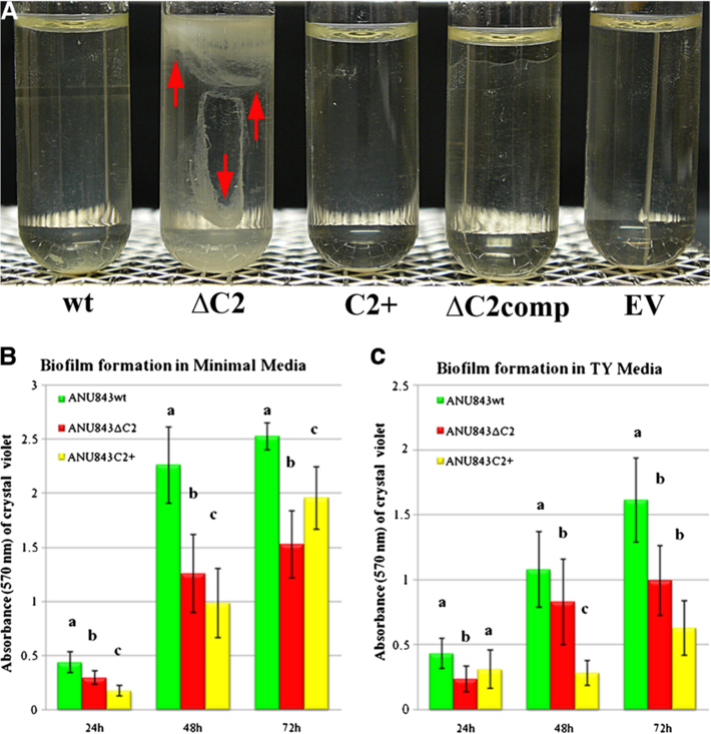
**Attachment on glass (A) and polystyrene plates (B-C).** (**A**) Ring formation at the glass-air-liquid interface after bacterial static growth*.* From left to right ANU843 (wild-type strain) and its derivatives ANU843ΔC2 (ΔC2), ANU843C2^+^ (C2+), ANU843ΔC2 complemented (ΔC2comp), and ANU843emptyvector (EV). Note the formation of a thick visible ring in ΔC2. (**B**-**C**) The data show absorbance values of CV-stained biofilms following growth in minimal media and TY at different times after inoculation. The culture media of the static culture was removed and differences in biofilm matrix development were measured by the intensity of CV staining. Before CV staining, the OD_600_ of the broth cultures were measured in a Microtiter Plate reader to verify that no differences in growth rate among the wells had occurred. Each datum point is the average of at least 20 wells. Error bars indicate the standard deviation. Each experiment was repeated at least three times. Values followed by the same letter do not differ significantly according to Fisher protected LSD test at P = 0.01. The degree of biofilm formation was significantly different among the strains tested, although these differences were less pronounced in the complex medium.

The ANU843ΔC2 mutant, defective in the synthesis of cellulase CelC2, flocculated heavily into large cell aggregates in YMB liquid culture, whereas this extensive formation of clumps was not observed in wild type ANU843 and flocculation was undetectable in the CelC2 overproducing derivative strain ANU843C2^+^ (Figure 2A). Furthermore, neither the *celC* complemented strain nor ANU843 carrying the empty vector showed extensive flocculation (Figure 3A). Settling of ANU843ΔC2 autoaggregated flocs at 1 x g assessed at 24 h resulted in a reduction of 67% in the OD_600_ of the upper part of the unshaken culture tubes as compared to the wild type. These flocs were completely dispersed after treatment with commercial cellulase (Sigma) for 2 h (Figure 2B-C). Aggregation of bacteria, usually mediated by cellulose micro fibrils, and dispersion of cell aggregates by exogenously added cellulase are highly suggestive that an excess of extracellular cellulose is produced [22].

Intensity of Congo red staining of ANU843ΔC2 and ANU843C2^+^ strains was greatly increased or reduced, respectively, as compared to the wild type and ANU843 carrying the empty vector (Figure 1D). Also, the intensity of Congo red staining of the *celC* complemented strain was reduced as compared to ANU843ΔC2, showing a complementation of this phenotype (Figure 1D).

The mean widths of line streak colonies for ANU843wt, ANU843ΔC2 and ANU843C2^+^ when grown on YMA plates (Figures 2D-F) were 13.0 ± 1.3, 5.0 ±1.0, and 6.7 ± 1.3 millimeters, respectively, and ANOVA statistics indicated that these differences in width were statistically significant (N = 12, p ≤ 0.03). A similar trend is represented in their mucoidy (Figure 2D-F, enlargement).

In addition, quantitative image analysis of fluorescence micrographs of cultures stained with the fluorochrome Calcofluor, which binds to β-linked glucans like cellulose, indicated that the extracellular micro fibrils associated with cells of the ANU843ΔC2 mutant were significantly longer than the average associated with wild-type (up to 15 μm vs. 8.34 μm standardized per cell, respectively), and that they were not detected in association with the ANU843C2^+^ derivative strain (Figure 2G-I).

Thus, the increase in flocculation, enzymatic treatment, Congo red staining, and estimated microfibril length observed in the ANU843ΔC2 mutant are all directly correlated with, hence most likely due to the overproduction of external cellulose micro fibrils.

### Cellulose micro fibrils involvement in biofilm formation and maturation

Among the molecules involved in biofilm development are different proteins and some exopolysaccharides, such as cellulose, which is a major component of the biofilm matrix of several bacterial species [30]. Cellulose-production defective mutants of different bacterial species have been shown to be impaired in biofilm development [36-38]. By testing biofilm formation on different abiotic substrata and examining attachment *in planta* with the host plant clover, we qualitatively and quantitatively compared the biofilm formation ability of the wild type strain ANU843 and its derivatives ANU843ΔC2 and ANU843C2^+^, based on methods previously described [3]. Different patterns of biofilm formation on both abiotic and biotic substrates were observed for the different strains.

Ring production of ANU843ΔC2 at the glass-liquid-air interface were thicker, more compact, and more easily dislodged from the glass surface compared to the other strains tested (Figure 3A, red arrows) probably due to the overproduction of external cellulose micro fibrils.

Growth of ANU843ΔC2 bacteria was slightly delayed and more flocculated than wild-type in microtiter wells containing YMB. The use of either TY or minimal media containing mannitol resulted in more clear-cut results, with no detectable differences in growth among the strains (data not shown), but showing statistically significant reduced biofilm formation for the mutants compared to the wild-type (Figure 3B-C). ANU843ΔC2 mutants exhibited a 30–50% decrease in biofilm formation in the microtiter plate assay (Figure 3B-C) in minimal media and TY. ANU843C2^+^ exhibited ca. 30–60% reduced biofilm formation compared to the wild-type strain 24 h and 48 h after the start of the experiment. However, the differences were less pronounced at 72 h.

The most striking difference in biofilm formation was found between the ANU843ΔC2 mutant and wild-type strains because prior to washing, the biofilms of the mutant appeared to be robust and compact. However, ANU843ΔC2 flocs were easily removed with each successive washing step, whereas almost all of the wild-type cells remained firmly attached to the plastic wells (image not shown).

**Figure 4:**
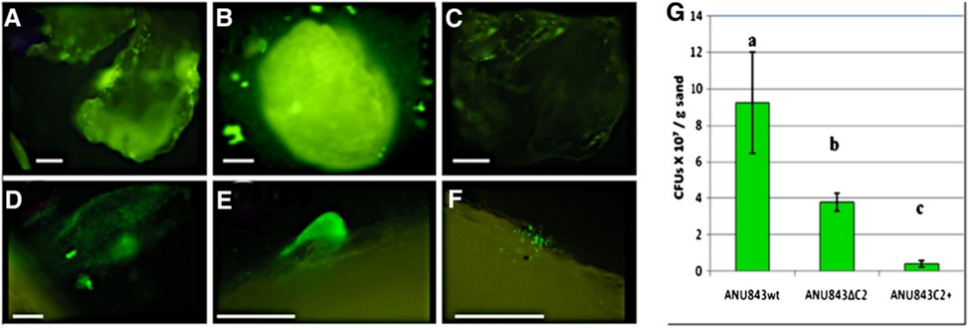
**Test for adhesion to sand (A-C) and biofilm formation on PVC (D-F) tabs of the studied strains marked with GFP.** The wild-type strain formed three-dimensional structures (**A**, **D**) whereas ANU843ΔC2 established microcolonies forming a layer that coated the surface (**B**, **E**), and ANU843C2^+^ barely adhered (**C**, **F**). Bar (**A**-**F**) 500 μm. (**G**) We also evaluated attachment quantitatively, by counting cfus of bacteria attached to sand grains. Error bars indicate the standard deviation. Each experiment was repeated three times. Values followed by the same letter do not differ significantly according to the Fisher protected LSD test at P = 0.01.

We also examined biofilm development on a sand substratum representing a more natural environment for these bacteria. PVC tabs were used to examine the three-dimensional structure of biofilms formed at the edges of inert surfaces, as well. A week after inoculation, biofilm growth on sand (Figure 4A-C) and on plastic tabs (Figure 4D-F) in static GFP-labeled rhizobia cultures was examined by fluorescence microscopy.

Significant differences were found regarding the adhesion capacities of the different strains to sand (Figure 4A-C, G), consistent with results reported for Figure 3.

Mutants were also tested for biofilm-forming ability on PVC tabs. Wild type ANU843 developed microcolonies, which progressed into a characteristic three-dimensional biofilm (Figure 4D). This biofilm morphology sharply contrasted with that produced by the ANU843ΔC2 mutant in which only thick, tightly appressed mounds of cells were apparent (Figure 4E). On the other hand, the three-dimensional biofilm structure of GFP-tagged ANU843C2^+^ was not detected. This strain only produced a 2-dimensional monolayer biofilm, with just a few bacteria attached to one another (Figure 4F). These results are consistent with results obtained in the sand assay and confirm the role of external cellulose micro fibrils in biofilm attachment and architecture. They further suggest that a *R. leguminosarum* bv. trifolii biofilm does not develop normally if cellulose micro fibril production is altered by elevated or diminished levels of CelC2 endoglucanase (or by the activity of the *celC* gene product).

### Cellulose micro fibrils involvement in biofilm formation and maturation *in planta*

**Figure 5:**
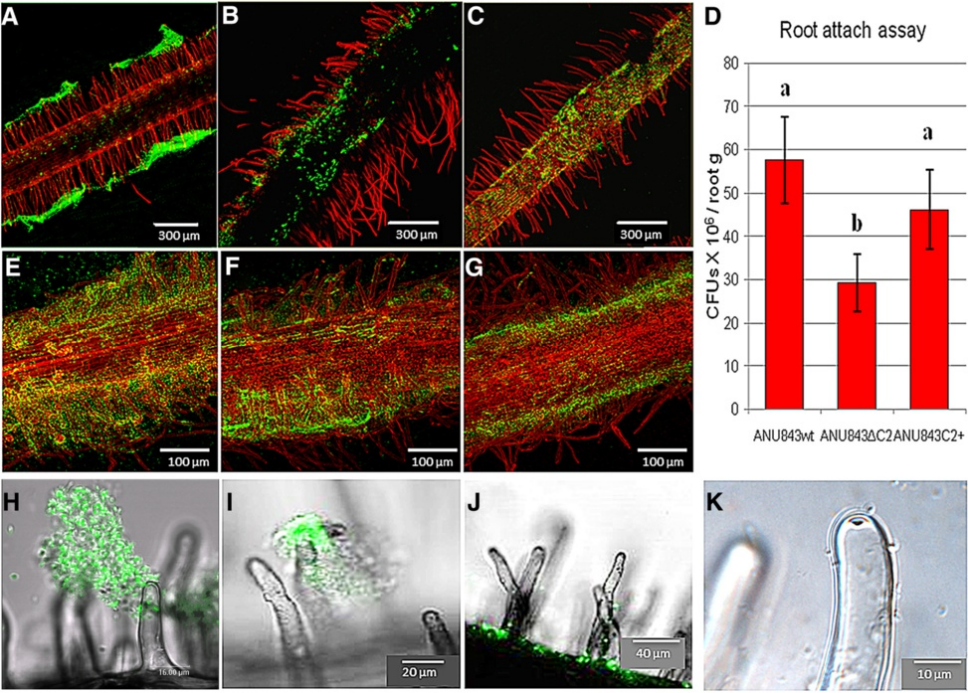
**Root attachment assays used to study the ability of rhizobia to form biofilms on*Trifolium repens*.** (**A-C**, **E-G**) Confocal laser scanning microscopy of propidium iodide-stained roots inoculated with gfp-tagged ANU843 and its derivatives showing biofilm formation along the root surface at different magnifications. (**D**) Number of colony-forming units (cfu) per gram of root tissue after washing and sonicating the roots. Each datum point is the average of at least 9 determinations. Error bars indicate the standard error from the mean. Root biofilms with either wild-type or *celC* mutant bacteria were harvested 72 h post-inoculation. Fluorescence (**H-J**) and phase-contrast (**K**) microcopy show root hair colonization in detail. The wild-type strain (**A**, **E**, **H**) forms three-dimensional biofilms that cover both root surface and root hairs forming distinct “caps” on the tip (H). In contrast, ANU843ΔC2 (**B**, **F**, **I**) establishes aggregates that cover the root irregularly and forms a thicker cap on the root hairs (**I**) whereas ANU843C2^+^ (**C**, **G**, **J**) appears to coat the root surface without cap formation (**J**). Nevertheless, sufficient adhesion of individual bacteria occurs on the tip to produce the hot (hole on the tip) phenotype (**K**).

We examined attachment and biofilm formation of *Rhizobium leguminosarum* bv. trifolii and its *celC* derivative strains on roots and root hair tips of the host plant *Trifolium repens* in N-free Fähraeus medium to extend the results found in the biotic surface tests. All strains colonized the root and attached especially the root surface (Figure 5). Even strain ANU843C2^+^ impaired in cellulose micro fibrils biosynthesis attached to plant roots, further confirming that bacterial cellulose does not act alone in plant root attachment.

In ANU843, the majority of root hairs in the growing root hair zone were covered with *R. leguminosarum* bv. trifolii cells growing in a three-dimensional biofilm (Figure 5A, E, H) whereas ANU843ΔC2 formed large aggregates that covered the root in an irregular manner (Figure 5B, F, I). In contrast, very few individual cells of the ANU843C2^+^ derivative strain were attached to root hairs (Figure 5C, G, J, K). Quantitative micro densitometry of GFP-dependent cell fluorescence (sum gray/mm root length) in Figures 5A, B and C indicated intensity values of 246, 64, and 34 (ratios of 7.2 : 1.9 : 1.0) for wild type, ANU843ΔC2 and ANU843C2^+^ strains, respectively.

There was attachment of individual GFP-labeled *R. leguminosarum* bv. trifolii ANU843 wild-type and strain ANU843ΔC2 bacterial cells, followed by “cap” formation, in which additional bacterial cells attach to bacteria that directly bind to root hairs (Figure 5A, E, H and B, F, I, respectively). However, ANU843ΔC2 cells more often clustered in discrete clumps on the root surface (Figure 5B, F) including both non-root hair and root hair epidermal cells (Figure 5I). In contrast, the biofilm caps on root hair tips were significantly reduced in frequency for the ANU843C2^+^ strain (Figure 5C, G, J, K). These results confirm that cellulose is not required for the initial, phase-I hapten-inhibitable lectin-mediated attachment to root hairs [1], although its production does intensify the three-dimensional Phase 1A cap biofilm formation [18]. The differences in pattern of root and root hair attachment by the cellulose mutants implies that the cellulose fibrils play a significant role in stability of the three-dimensional root hair “cap” biofilm.

Clover roots were also harvested, washed, and sonicated for colony counts. For strain ANU843, an average of 5.8 x 10^7^ rhizobia cells per mg root fresh weight were attached to the root (Figure 5D). By contrast, only 3 x 10^7^ ANU843ΔC2 cells per mg root fresh weight attached to clover roots, showing that, as occurred in the abiotic surface experiments, the unstable aggregates on surfaces are easily removed (Figure 5D). Regarding ANU843C2^+^, 4.6 x 10^7^ cells per mg of root fresh weight were attached, showing that the stunted three-dimensional structures led to slightly loose bacterial attachment. However, no significant differences with respect to ANU843 were detected in terms of CFU/mg. It remains to be determined if this difference is due to loss of the recombinant plasmid in the absence of the selector marker *in planta*, or to the possible regulation of bacterial cellulose production by the plant, perhaps by inhibiting CelC2 activity or by repressing expression of the *celC* gene.

**Figure 6:**
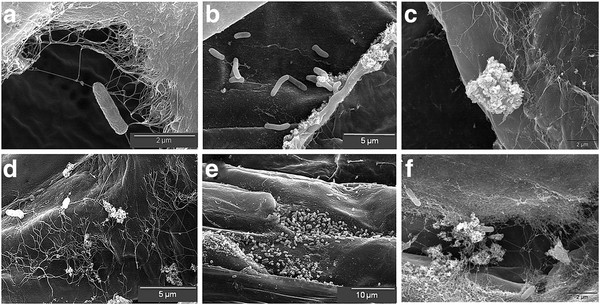
**Scanning Electron Microscopy (SEM) of clover roots inoculated with the strains under study: (A, D) ANU843 wild-type strain, (B, E) ANU843C2**^**+ **^**and (C, F) ANU843ΔC2.**

We finally analyzed in detail by scanning electron microscopy the adhesion capacity of the strains under study to the root surface. Figure 6 shows that most wild type cells are attached to the root surface and surrounded by cellulose micro fibrils (Figure 6A, D). Differential counts of 17 micrographs indicated a frequency of approximately 82% in this category. While samples inoculated with the CelC2 overproducing strain had fewer bacteria coated with cellulose micro fibrils (Figure 6B, E) occurring in an approximate frequency of 50%. However, it was not possible to make reliable ANU843ΔC2 cells count due to the higher presence of cellulose micro fibrils (Figure 6C, F).

## Discussion

Cellulose production in bacteria is of potential economic interest because of its impact in medical settings and in the paper and food industry [30]. In addition, cellulose biosynthesis in a wide variety of bacteria has increased the possibility for the elucidation of regulation and molecular mechanisms of cellulose biosynthesis, which still remains largely unknown. The role of cellulose in attachment and biofilm formation during the interaction of the bacteria with the environment and its hosts confers further importance to this polymer as a potential target to design methods against harmful biofilm proliferation. In this work, we report how the alterations in the levels of endogenous cellulase contribute to biofilm architecture.

Although Congo red can also bind outer membrane proteins in some animal pathogens [39], several authors have noted that among *Rhizobium* spp., staining with this dye correlated with the cellulose content in the bacterial cultures [16,40]. The significant reduction in Congo red binding to engineered strains of *Rhizobium* impaired in cellulose production (Figure 1D) suggests that these bacteria do not produce other substances that strongly bind Congo red and contribute to their red colony pigmentation. Both the binding to Congo red in diverse species of the family *Rhizobiaceae* and the presence of genes encoding cellulose synthases in their sequenced genomes strongly suggest that the ability to synthesize cellulose is fairly common in this taxonomic group of plant-associated bacteria. Several lines of evidence further support that cellulase activity is commonly involved in the cellulose production pathway used by these species: i) the existence of at least a cellulase-encoding gene associated to a glycosil transferase-encoding gene in all *Rhizobiaceae* species with accessible data in GeneBank, ii) the presence of the *celABC* operon within *Rhizobiaceae,* and iii) the high degree of conservation of CelC cellulase-encoding genes between *Rhizobium* species [35].

The ability of most legume root-nodulating microorganisms to synthesize cellulose implicates the importance of this polymer in their eco-physiology. Furthermore, although the inactivation of cellulose biosynthesis in *R. leguminosarum* bv. trifolii does not affect the ability to nodulate clover in controlled laboratory conditions [16], the ability of rhizobia to adhere firmly to a substrate using cellulose micro fibrils facilitates host root colonization [1,8]. Moreover, it is very possible that cellulose micro fibril-mediated firm adhesion during legume host root colonization is important under natural conditions in the rhizosphere (or rhizospheric soil), where bacteria have to compete for survival and colonization to successfully gain access to plant carbon sources.

During the course of these studies on the CelC2 protein, we detected by Congo Red and Calcofluor staining microscopy and enzymatic treatment that the *celC* over-expression derivative strain lost the ability to make extracellular cellulose micro fibrils, and that the extracellular micro fibrils of the *celC* knockout mutant were significantly longer than those seen in the wild type parent (Figure 2) suggesting that *R. leguminosarum* bv. trifolii cellulase CelC2 is involved in determining the longitude of cellulose extracellular micro fibrils.

Ausmees *et al.*[16] found that cloned genes involved in cellulose biosynthesis have similar homology and the same organization in *R. leguminosarum* bv. trifolii strain R200 and *A. tumefaciens*. These authors identified these genes by Tn*5* mutagenesis followed by screening with Calcofluor staining for mutants showing less ability to synthesize cellulose. They obtained *celA*, *celB,* and *celE* mutants but not *celC*, consistent with our finding that a *celC* knock-out mutant overproduces external cellulose micro fibrils. Therefore, we conclude that the *celC* gene is involved in the formation and elongation of cellulose micro fibrils. It is likely that cellulose oligomers synthesized by CelA elongate indefinitely in the absence of the CelC endoglucanase, producing very long micro fibrils that entangle ANU843ΔC2 mutant bacterial cells into very large aggregates causing them to flocculate and settle in liquid medium cultures (Figures 2A and 3A). By contrast, an excess of endoglucanase activity leads to an uncontrolled degradation of CelA-synthesized cellulose oligomers, preventing their transport and subsequent maturation into microcrystalline micro fibrils that extend outside the cell (Figure 2I). The fluorescence intensity of these CelC2-degraded oligomer products suggest that they may concentrate at both cell poles (Figure 2G-I inserts).

In the model proposed for the synthesis of cellulose in plants [41], CesA protein is a polymerase that catalyzes ß-1,4-glucan chain elongation by transferring UDP-glucose moieties to the sitosterol-ß-sitosterol-glucoside intermediate, forming cellodextrins. The other protein that has been proposed to participate in this process is Korrigan (Kor) cellulase, which may act by releasing the sitosterol-ß-glucoside from the newly synthesized cellulose polymer chain. How the final cellulose chain is formed, what other molecule(s) intervene in this step, the mechanism of its export and components of its anchoring apparatus on the cell surface all remain unknown. However, in the model for cellulose synthesis in bacteria [32], CelA activity adds glucose monomers from the UDP-glucose substrate to the intermediate lipid-glucose to form lipid-glucose_x_ (x = 2–4 glucose). Bacterial CelA and plant CesA are both glycosyl transferases, whereas CelC and Korrigan present a glycosyl hydrolase domain. According to Matthysse *et al.*[32], bacterial CelC cellulase may act in this case as a translocase, by incorporating the lipid-linked oligosaccharide into the cellulose polymer chain being formed. According to our findings, the role of CelC cellulase in cellulose biosynthesis is similar to the role that has been proposed for Korrigan cellulase. This CelC cellulase may catalyze the hydrolysis reaction, in which cellotriose are released from the lipid-glucose_4_ intermediary, thus providing the substrate for the translocase to transfer it to the internal growing point of another lipid-intermediate, thereby elongating the cellulose microfibril product by 3 glucose units at one time.

This function for the *celC* gene is distinctly different from its established role as an endoglucanase involved in the infection process [28]. The fact that core *celC* has both colonization and infection functions, *i.e.*, in cellulose biosynthesis and independently as a hydrolytic enzyme that creates the portal of *Rhizobium* entry into the host root hair and their liberation from infection threads into the symbiosomes within nodule cells, implies the likely existence of two different sets of control mechanisms, functional designs and target cellular locations. Furthermore, the possible role in cellulose biosynthesis of cellulase CelC1, that it is not involved in plant root hair tip erosion [33], has not been yet characterized. We propose that cellulose production may reflect an earlier evolutionary development because this property is encoded by an operon common to all nodulating rhizobia, including *Burkholderia* and *Cupriavidus* strains, the so-called β-rhizobia [42], whereas the cellulase *celC* gene function for the infection may develope later.

Previous results have shown that Tn*5* mutation in *nodC* diminishes extracellular microfibril production by ANU843 on white clover roots [43] and that *Rhizobium* common *nod* genes are required for biofilm formation [44]. Nevertheless, the role of cellulose in *Rhizobium* biofilm establishment has not been fully recognized. To our knowledge, this is the first time that *Rhizobium* mutants either lacking or over-expressing cellulase have been analysed with respect to cellulose production and biofilm formation on both abiotic surfaces and the root epidermis.

Our results show that biofilm formation ability was markedly reduced in ANU843ΔC2 and ANU843C2^+^ strains not only on abiotic surfaces (PVC, microtiter plate assays, and sand Figures 3 and 4), but also on plant roots (Figure 5). Since ANU843C2^+^ is impaired to produce external micro fibrils, it cannot tightly bind to the substrate preventing subsequent mature biofilm formation. On the other hand, it seems that ANU843ΔC2 cells, which produced more cellulose micro fibrils, tended to associate themselves more than to the plastic surfaces. However, no such caps are observed on sand grains. Since the PVC surface is hydrophobic while sand surface is hydrophilic, it might be possible that adhesion to each of these surfaces has different physicochemical constraints, and cellulose might play different roles in these scenarios.

Microtiter well, sand attachment and microscopy assays of GFP-tagged bacterial cultures confirmed the role of cellulose in affecting biofilm structure. We propose that cellulase CelC is associated with cellulose cleavage and processing, and that extracellular cellulose micro fibrils, but not in excess, are needed to build the three-dimensional structure of a mature biofilm of *R. leguminosarum* bv. trifolii on plastic, sand and white clover host root surfaces.

## Conclusions

Taking all together, these results show that *celC* gene deletion lead to an increase of external cellulose micro fibrils production and that its overexpression results in the opposite effect, elucidating the role of this gene in the *celABC* cellulose biosynthesis operon. Furthermore, the investigation of the mutants ability to form biofilms in different surfaces indicates that *celC* gene suitable expression is necessary for biofilm formation and maturation in *Rhizobium*. All this data confirm that *Rhizobium* cellulose is an essential component of the biofilm polysaccharidic matrix architecture and that either an excess or a defect of this “building material” seem to collapse the biofilm structure. These results position cellulose hydrolitic enzymes as excellent anti-biofilm candidates.

## Methods

### Bacterial strains, plasmids and growth conditions

Representative type strains of bacteria that form legume root-nodule symbioses and *Rhizobium leguminosarum* bv. trifolii strains used in this study are listed in Additional file 1 and Additional file 4 respectively. The former share the ability to form 1–5 mm colonies after 5–7 days on Yeast Mannitol Agar [45]. *R. leguminosarum* strains were also grown at 28°C in Triptone Yeast extract (TY) [46], Yeast extract Mannitol Broth (YMB) [47] or minimal medium containing 1% mannitol (MM) [48] or 0.5% inositol (BINOS) [49] as the carbon source. *Escherichia coli* S17.1 strain was grown at 37°C in LB medium [50]. These media were supplemented with kanamycin (50 μg/ml) or tetracycline (10 μg/ml) as required. Plasmid pHC60 [51] was introduced via biparental mating to yield GFP-tagged bacteria [34].

### Cellulose detection assays

Cellulose production was assayed by growing the strains in YMA plates containing 25 mg/l Congo red for 7 days to stain for cellulose, followed by background-subtracted quantitation of colony red luminosity by image analysis. For direct microscopy visualization of cellulose micro fibrils, cells grown on YMA plates were suspended in 0.025% Calcofluor (Sigma), placed onto Teflon printed slides and examined by fluorescence microscopy. Flocculation assays were performed basically as described in [52]. Overnight cultures were adjusted to an OD_600_ of 0.6 by dilution with YMB medium. Three-ml aliquots of cultures were incubated in 10 ml standing tubes at room temperature, and the OD_600_ of the upper parts of the cultures was measured 24 h after settling of flocs at 1 x g. These tests were performed in triplicate.

### Amplification and sequencing of the *Rhizobium leguminosarum* bv. trifolii ANU843 *celABC* operon

The region upstream *celC* gene containing *celA* and *celB* genes in *R. leguminosarum* bv. trifolii was amplified and sequenced by using primers designed in this work (Additional file 5) from conserved gene sequences available in databases. DNA was extracted according to [53]. PCR DNA amplification and agarose DNA electrophoresis were performed by using standard procedures [50]. The sequence reaction was performed on an ABI377 sequencer (Applied Biosystems Inc., USA) using a Big Dye terminator v3.0 cycle sequencing kit as supplied by the manufacturer. The *celABC* sequences obtained were compared with those present in other bacterial genomes available in GenBank using the BLASTN program, while BLASTP was employed to compare the encoding proteins.

### Determination of biofilm formation

Ring formation at the glass-air-liquid interface were qualitatively scored after 3 to 5 days of bacterial growth in 5 ml of TY medium in glass tubes shaken at 180 rpm in an orbital shaker followed by static growth for 15 days*. In vitro* biofilms were established as described earlier [3]. Basically, to prepare the initial inocula, GFP-tagged bacteria were grown in the corresponding liquid medium for 2 days (extrapolated OD_600_ of approximately 2.0). They were then washed and diluted in the same medium to OD_600_ = 0.2 (ca. 1x10^7^ cells/ml) for all subsequent assays. Bacterial attachment to PVC plates was assayed by pipetting 100 μl of this culture into individual PVC wells in a 96-well plate (Falcon 3911, Becton Dickinson, Franklin Lakes, NY). The plates were sealed with parafilm and incubated at 28°C. At defined times, the growth media and unbound cells were removed, and attached bacteria were quantified by staining them with 0.3% (wt/vol) Crystal Violet (CV) (Sigma) for 10 min. After the excess dye was washed away and the wells allowed to dry, the remaining dye was solubilized with 80% ethanol-20% acetone and quantified by measuring the absorbance at 570 nm in an ASYS (Biochrom, UK) Microtiter Plate reader (Model No. UVM340).

Biofilm formation on abiotic surfaces was assayed by transferring 0.5 ml of each strain’s TY culture to individual wells of a 24-well Costar PS (polystyrene) plate containing either a PVC tab or 1ml of sterile sand substrata. After 1 week at 28°C, either the tabs or an alicuot of the sand particles previously washed with sterile water were examined by epifluorescence microscopy to evaluate the two- and three-dimensional architectures of the biofilms. In parallel, equal amounts of sand particles (aprox. 100 μl) were taken with a pipette harbouring a cut edge, weighted, and washed three times with sterile water. The cells were finally suspended in 1 ml of TE buffer (pH 7.0), dissociated at 25°C by 2 sonication pulses (37 kHz, 30 W) of 1 min each with pause time of 1 min between the pulses, in an Elmasonic (Singen, Germany) sonication bath and quantified by counting CFUs (normalized to the amount of sand weighted).

Root biofilms were prepared by dipping one-week-old seedling roots into a *Rhizobium* suspension of OD_600_ = 0.02 (ca. 1 x 10^6^ cells/ml) in Fähraeus N-free medium for 5 min. The seedlings were then grown on a Whatman paper support placed inside glass tubes containing 20 ml Fähraeus N-free medium. The colonization of the bacteria to the root and the root hairs was evaluated using confocal microscopy at different time points. To count the number of colonized rhizobia three days after inoculation, the roots were washed three times with sterile water under vigorous shaking to remove loosely associated cells, then dried at room temperature for one minute on sterile Whatman paper to eliminate the excess of water, and weighed. The dried roots were immersed in 1 ml of sterile TE buffer and sonicated as it was described above to release attached cells, which were quantified by colony counting (normalized to the roots weight). For each strain tested, we counted rhizobial attachment to at least four roots. All binding experiments were repeated at least three times.

### Microscopy

Calcofluor-stained bacteria were examined using epifluorescence optics (100 w HBO light source, 365/395/420 nm filter set) in a Zeiss universal microscope. Scanning electron microscopy (SEM) was performed as previously described [33], except that the samples were coated with osmium instead of gold.

GFP-tagged rhizobia growing on sand particles or plastic tabs were aseptically removed from the wells, and placed into the wells of depression slides, topped with a covers lip, and examined by confocal microscopy.

Bacterial attachment to the root hairs was observed by laser scanning confocal microscopy with a Leica microscope using 488-nm argon laser excitation and a 500-nm long-pass emission filter to allow observation of GFP-labeled bacteria, and phase contrast microscopy to observe root hairs. Images were processed into loss-less montages using Leica confocal software. Biofilms of GFP-labelled bacteria on roots were examined on a Zeiss LSM510 confocal microscope. Roots were stained with 10 μM of propidium iodide (Sigma). Projections were made from adjusted individual channels in the image stacks using Zeiss LSM510 imaging software.

### Digital image analysis

Center for Microbial Ecology Image Analysis (CMEIAS) software was used to measure the intensity of Congo red uptake and mean diameter of bacterial colonies, the calcofluor-fluorescent extracellular micro fibril length normalized to cell number, the % substrata coverage by the biofilms, and the *in situ* local density of GFP-tagged bacteria colonized on roots [54-56].

## Competing interests

The authors declare that they have no competing interests.

## Authors' contributions

MR, EMM, AMH and PFM participated in the design of the study; MR, LR, FD, and PFM performed research and data adquisition; MR, AMH, FD, and PFM. analyzed data; MR drafted the manuscript and JIJZ, RR, FD, EV, AMH, and PFM revised it critically for important intellectual content and gave final approval of the version to be published.

## Supplementary Material

Additional file 1 **Intensity of Congo red uptake by colonies grown on YMA of representative type strains of bacteria that form root-nodule symbioses with legumes and related, indicative of cellulose production.** +++/++/+: positive to different degrees; w: weakly positive. “Classical rhizobia” species are in bold. *Species whose nodulation capacity has not been described, including those genera traditionally considered as “rhizobia”. Click here for file

Additional file 2 **Putative rhizobial operons involved in cellulose biosynthesis located in the sequenced genome of rhizobia.** *Data from GenBank. Click here for file

Additional file 3 **Percentage of similarity between the proteins CelA, CelB and CelC of *Rhizobium leguminosarum*bv. trifolii ANU843 and those located in the sequenced genome of rhizobia and related plant-symbionts.** Data obtained using BLASTP program. Click here for file

Additional file 4 **Strains of *Rhizobium leguminosarum *bv. trifolii used in this study.** Click here for file

Additional file 5 Primers used in this study. Click here for file
