# Supplementary material for: Role of Rhizobium endoglucanase CelC2 in cellulose biosynthesis and biofilm formation on plant roots and abiotic surfaces
Source: Microb Cell Fact. 2012 Sep 12;11:125. doi: 10.1186/1475-2859-11-125 (PMC3520766; doi:10.1186/1475-2859-11-125)
Supplement: Additional file 1 — Intensity of Congo red uptake by colonies grown on YMA of representative type strains of bacteria that form root-nodule symbioses with legumes and related, indicative of cellulose production. +++/++/+: positive to different degrees; w: weakly positive. “Classical rhizobia” species are in bold. *Species whose nodulation capacity has not been described, including those genera traditionally considered as “rhizobia”. [file 1475-2859-11-125-S1.doc]

**Additional file 1**. Intensity of Congo red uptake by colonies grown on YMA of representative type strains of bacteria that form root-nodule symbioses with legumes and related, indicative of cellulose production. +++/++/+: positive to different degrees; w: weakly positive. “Classical rhizobia” species are in bold. *Species whose nodulation capacity has not been described, including those genera traditionally considered as “rhizobia”.

| GenUS | Type strain | legume HOST | Congo Red Uptake |
| --- | --- | --- | --- |
| *Rhizobium* | ***R . cellulosilyticum* ALA10B2 ^T^** | *Medicago* | +++ |
|  | ***R. etli* CFN 42^T^** | *Phaseolus* | +++ |
|  | ***R. galegae* ATCC 43677^T^** | *Galega* | +++ |
|  | ***R. gallicum* R602sp^T^** | *Phaseolus* | + |
|  | ***R. giardinii* H152^T^** | *Phaseolus* | +++ |
|  | ***R. hainanense* I66^T^** | *Desmodium* | + |
|  | ***R. huautlense* SO2^T^** | *Sesbania* | + |
|  | ***R. indigoferae* CCBAU 71042^T^** | *Indigofera* | + |
|  | ***R. leguminosarum* ATCC10004^T^** | *Pisum* | + |
|  | ***R. leguminosarum* bv.** trifolii **ANU843** | *Trifolium* | ++ |
|  | ***R. loessense* CCBAU 7190B^T^** | *Astragalus* | ++ |
|  | ***R. lusitanum* P1-7^T^** | *Phaseolus* | + |
|  | ***R. mongolense* USDA 1844^T^** | *Medicago* | +++ |
|  | ***R. radiobacter* ATCC 19358^T^** | * | + |
|  | ***R. rhizogenes* ATCC 11325^T^** | *Phaseolus* | ++ |
|  | ***R. sullae* IS123 ^T^** | *Hedysarum* | + |
|  | ***R. rubi* ATCC 13335^T^** | *** | ++ |
|  | ***R. phaseoli* ATCC 14482^T^** | *Phaseolus* | + |
|  | ***R. pisi* DSM 30132^T^** | *Pisum* | ++ |
|  | ***R. tropici* CIAT 899 ^T^** | *Phaseolus* | + |
|  | ***R. yanglingense* CCBAU 71623** | *Amphicarpaea* | + |
| *Ensifer* | ***E. fredii* LMG 6217^T^** | *Glycine* | + |
|  | ***E. kostiense* LMG 19227 ^T^** | *Acacia* | w |
|  | ***E. meliloti* ATCC 9930 ^T^** | *Medicago* | + |
|  | ***E. meliloti* 1021** | *Medicago* | w |
|  | ***E. medicae* LMG1037 ^T^** | *Medicago* | +++ |
|  | ***E. medicae* WSM419** | *Medicago* | + |
|  | ***E. morelense* Lc04 ^T^** | *** | ++ |
|  | ***E. saheli* LMG7837 ^T^** | *Acacia* | ++ |
|  | ***E. terangae* LMG6463 ^T^** | *Acacia* | + |
|  | ***E. xinjiangense* LMG17930 ^T^** | *Glycine* | + |
| *Mesorhizobium* | ***M. amorphae* ACCC 19665 ^T^** | *Amorpha* | ++ |
|  | ***M. chacoense* Pr5 ^T^** | *Prosopis* | w |
|  | ***M. ciceri* USDA 3383 ^T^** | *Cicer* | + |
|  | ***M. huakuii* USDA 4779 ^T^** | *Astragalus* | + |
|  | ***M. loti* ATCC 33669 ^T^** | *Lotus* | + |
|  | ***M. mediterraneum* USDA 3392^T^** | *Cicer* | ++ |
|  | ***M. plurifarium* LMG 7836^T^** | *Acacia* | + |
|  | ***M. septentrionale* HAMBI 2582 ^T^** | *Astragalus* | + |
|  | ***M. tianshanense* LMG 18976 ^T^** | *Sophora* | ++ |
|  | ***M. temperatum* HAMBI 2583 ^T^** | *Astragalus* | w |
| *Phyllobacterium* | *P. trifolii* pETPO2 ^T^ | *Trifolium* | + |
| *Bradyrhizobium* | ***B. betae* pl7Hg^T^** | *** | + |
|  | ***B. canariense* BTA1^T^** | *Chamaecytisus* | +++ |
|  | ***B. elkanii* LMG 6134^T^** | *Glycine* | + |
|  | ***B. japonicum* LMG 6138^T^** | *Glycine* | + |
|  | ***B. liaoningense* LMG 18230^T^** | *Glycine* | + |
|  | ***B. yuanmingense* LMG 21827^T^** | *Lespedeza* | + |
| *Azorhizobium* | ***A. caulinodans* ORS 571^T^** | *Sesbania* | + |
| *Devosia* | *D. neptuniae* J1^T^ | *Neptunia* | ++ |
| *Ochrobactrum* | *O. lupini* LUP21^T^ | *Lupinus* | + |
|  | *O. cytisi* LMG18957^T^ | *Cytisus* | + |
