# Supplementary material for: Role of Rhizobium endoglucanase CelC2 in cellulose biosynthesis and biofilm formation on plant roots and abiotic surfaces
Source: Microb Cell Fact. 2012 Sep 12;11:125. doi: 10.1186/1475-2859-11-125 (PMC3520766; doi:10.1186/1475-2859-11-125)
Supplement: Additional file 2 — Putative rhizobial operons involved in cellulose biosynthesis located in the sequenced genome of rhizobia. *Data from GenBank. [file 1475-2859-11-125-S2.doc]

**Additional file 2**. Putative rhizobial operons involved in cellulose biosynthesis located in the sequenced genome of rhizobia. *Data from GenBank.

| Organism | Cellulose operon | Locus Tag* |
| --- | --- | --- |
| *Rhizobium leguminosarum* bv. trifolii WSM1325 | *celABC* | Rleg1293, Rleg1294, Rleg1295 |
|  | *celIJK* | Rleg43342, Rleg43343, [Rleg4334](http://www.ncbi.nlm.nih.gov/sites/entrez?db=gene&cmd=search&term=8015915&RID=YV7HYHDS014&log$=geneexplicitprot&blast_rank=5) |
| *Rhizobium leguminosarum* bv. trifolii WSM2304 | *celABC* | Rleg21204, Rleg21205, Rleg21206 |
|  | *celIJK* | Rleg24003, Rleg24004, [Rleg24005](http://www.ncbi.nlm.nih.gov/sites/entrez?db=gene&cmd=search&term=6982775&RID=YV7HYHDS014&log$=geneexplicitprot&blast_rank=6) |
| *Rhizobium leguminosarum* bv. *viciae* 3841 | *celABC* | RL1646, RL1647, RL1648 |
|  | *celIJK* | RL0079, RL0080, RL0081 |
| *Rhizobium etli* CFN42 | *celABC* | RHECH01542, RHECH01543, RHECH01544 |
|  | *celIJK* | RHECH00070, RHECH00071, RHECH00072 |
| *Rhizobium etli* CIAT652 | *celABC* | RHECIATCH0001610, RHECIATCH0001611, RHECIATCH0001612 |
|  | *celIJK* | RHECIATCH0000074, RHECIATCH0000075, RHECIATCH0000076 |
| *Rhizobium sp.* NGR234 | *celABC* | NGRb15360, NGRb15370, NGRb15380 |
|  | *celIJK* | NGRb17870NGRb17890NGRb17880 |
| *Agrobacterium tumefaciens* C58 | *celABC* | ATU3309, ATU08, ATU3307 |
|  | *celIJK* | ATU3314, ATU3313, ATU3312 |
| *Agrobacterium radiobacter* K84 | *celABC* | Arad9974, Arad9975, Arad9976 |
|  | *celIJK* | Arad7615, Arad7617, [Arad7618](http://www.ncbi.nlm.nih.gov/sites/entrez?db=gene&cmd=search&term=7365476&RID=YV7HYHDS014&log$=geneexplicitprot&blast_rank=19) |
| *Ensifer meliloti* 1021 | *celIJK* | SMb20460, SMb20461, SMb20462 |
| *Ensifer medicae* WSM419 | *celABC* | Smed5208, Smed5209, Smed5210 |
|  | *celIJK* | Smed3671, Smed3670, [Smed3669](http://www.ncbi.nlm.nih.gov/sites/entrez?db=gene&cmd=search&term=5318066&RID=YV7HYHDS014&log$=geneexplicitprot&blast_rank=28) |
| *Mesorhizobium loti* MAFF303099 | *celIKJ* | mll7871, mll7872, mll7873 |
