# Supplementary material for: Role of Rhizobium endoglucanase CelC2 in cellulose biosynthesis and biofilm formation on plant roots and abiotic surfaces
Source: Microb Cell Fact. 2012 Sep 12;11:125. doi: 10.1186/1475-2859-11-125 (PMC3520766; doi:10.1186/1475-2859-11-125)
Supplement: Additional file 3 — Percentage of similarity between the proteins CelA, CelB and CelC of Rhizobium leguminosarumbv. trifolii ANU843 and those located in the sequenced genome of rhizobia and related plant-symbionts. Data obtained using BLASTP program. [file 1475-2859-11-125-S3.doc]

**Additional file 3**. Percentage of similarity between the proteins CelA, CelB and CelC of *Rhizobium leguminosarum* bv. trifolii ANU843 and those located in the sequenced genome of rhizobia and related plant-symbionts. Data obtained using BLASTP program.

| **STRAIN** | **CelA** | **CelB** | **CelC** |
| --- | --- | --- | --- |
| *Rhizobium leguminosarum bv.* trifolii ANU843 | 100% | 100% | 100% |
| *Rhizobium leguminosarum bv.* trifolii WSM2304 | 100% | 100% | 98% |
| *Rhizobium leguminosarum bv.* trifolii WSM1325 | 98% | 100% | 86% |
| *Rhizobium leguminosarum bv.* trifolii R201 | 100% | 100% | 89% |
| *Rhizobium leguminosarum bv. viciae* 3841 | 96% | 100% | 87% |
| *Rhizobium etli* CIAT 652 | 96% | 90% | 90% |
| *Rhizobium etli* CFN42 | 97% | 89% | 90% |
| *Rhizobium* sp. NGR234 | 78% | 52% | 59% |
| *Agrobacterium tumefaciens* C58 | 73% | 53% | 56% |
| *Agrobacterium radiobacter K84* | 86% | 70% | 74% |
| *Ensifer medicae* WSM419 | 78% | 50% | 58% |
| *Methylobacterium nodulans* ORS 2060 | 65% | 30% | 41% |
| *Azospirillum* sp. B510 | 54% | 25% | 41% |
