# Supplementary material for: Role of Rhizobium endoglucanase CelC2 in cellulose biosynthesis and biofilm formation on plant roots and abiotic surfaces
Source: Microb Cell Fact. 2012 Sep 12;11:125. doi: 10.1186/1475-2859-11-125 (PMC3520766; doi:10.1186/1475-2859-11-125)
Supplement: Additional file 4 — Strains of Rhizobium leguminosarum bv. trifolii used in this study. [file 1475-2859-11-125-S4.doc]

**Additional file 4.** Strains of *Rhizobium leguminosarum* bv. trifolii used in this study.

| Strain | | Description | Source or reference |
| --- | --- | --- | --- |
| ANU843 | Wild-type Nod^+^ Fix^+^ | |  |
| ANU843ΔC2 | Wild-type containing a 361-bp deletion in *celC* gene | |  |
| ANU843ΔC2comp | ANU843ΔC2 containing plasmid pJZC2, carries *celC*; Km^r^ | |  |
| ANU843EV  ANU843C2^+^ | Wild-type containing the empty vector pBBR1MCS-2; Km^r^  Wild-type containing plasmid pJZC2, carries *celC*; Km^r^ | |  |
| ANU843GFP | Wild type containing pHC60 for gfp expression; Tc^r^ | |  |
| ANU843GFPΔC2 | ANU843ΔC2 containing pHC60 for gfp expression; Tc^r^ | | This work |
| ANU843GFPC2^+^ | ANU843C2^+^ containing pHC60 for gfp expression; Km^r^, Tc^r^ | |  |
