# Supplementary material for: Role of Rhizobium endoglucanase CelC2 in cellulose biosynthesis and biofilm formation on plant roots and abiotic surfaces
Source: Microb Cell Fact. 2012 Sep 12;11:125. doi: 10.1186/1475-2859-11-125 (PMC3520766; doi:10.1186/1475-2859-11-125)
Supplement: Additional file 5 — Primers used in this study. [file 1475-2859-11-125-S5.doc]

**Additional file 5.** Primers used in this study

| OcelF2 | 5´ CGATCTTCCTGTTTGCGCCGC 3´ |
| --- | --- |
| CelCS2F | 5´ AGCCGCTGATCGCCGGCC 3´ |
| OcelF3b | 5´ TGAGCAGCATGCCGAACCA 3´ |
| OcelR1 | 5´ GGTCGTTCCGCTGGCCCTGG 3´ |
| OcelR3 | 5´ CGCCGCCAACTGGCTGTCGA 3´ |
| CelCS1R | 5´ CCTGCAGACCCAGCTCATC 3´ |
| CelCS2R | 5´ CATCATCCAGCGCGGCCT 3´ |
| OcelF7 | 5´ GAATACGGCTCCGGTTCAGCG 3´ |
| R4 | 5´ CAGATAGACCGACCATGACC 3´ |
| R2 | 5´ TGCAATCGGTGCGGTGGCG 3´ |
| celB1R | 5´ GCCGCTCGCCGGACATGTCG 3´ |
| celC3F | 5´ GGCGTGGAACTGTTGAAGACG 3´ |
| ocelB1F | 5´ CGGTGACGGATGCGCAGACG 3´ |
| ocelF5 | 5´ gcggctggaacctcatctacct 3´ |
